# Nanoparticle corona artefacts derived from specimen preparation of particle suspensions

**DOI:** 10.1038/s41598-020-62253-y

**Published:** 2020-03-24

**Authors:** Martha Ilett, Omar Matar, Faith Bamiduro, Sergio Sanchez-Segado, Rik Brydson, Andy Brown, Nicole Hondow

**Affiliations:** 1 School of Chemical and Process Engineering, University of Leeds, Leeds, UK; 2 Department of Chemical and Environmental Engineering, Technical University of Cartagena, Campus Muralla del Mar C/Doctor Fleming s/n, Cartagena, 30202 Murcia, Spain

**Keywords:** Nanoscale materials, Nanoparticles

## Abstract

Progress in the implementation of nanoparticles for therapeutic applications will accelerate with an improved understanding of the interface between nanoparticle surfaces and the media they are dispersed in. We examine this interface by analytical scanning transmission electron microscopy and show that incorrect specimen preparation or analysis can induce an artefactual, nanoscale, calcium phosphate-rich, amorphous coating on nanoparticles dispersed in cell culture media. We report that this ionic coating can be induced on five different types of nanoparticles (Au, BaTiO_3_, ZnO, TiO_2_ and Fe_2_O_3_) when specimen preparation causes a significant rise in pH above physiological levels. Such a pH change reduces ionic solubility in the suspending media to permit precipitation of calcium phosphate. Finally, we demonstrate that there is no indication of a calcium-phosphorus-rich coating on BaTiO_3_ nanoparticles suspended in culture media when prepared without alteration of the pH of the suspending media and imaged by cryo-STEM. Therefore we recommend that future reports utilising nanoparticles dispersed in cell culture media monitor and report the pH of suspensions during sample preparation.

## Introduction

There is increasing evidence and understanding of the potential therapeutic benefits of using nanotechnology within medicine^1,2^. A wide range of nanoparticles have been studied and shown promise in applications such as drug delivery, medical imaging and cancer therapy^3–6^. Typically such nanomedical studies require nanoparticles to be dispersed in biological fluids. The complex nature of these fluids means that an understanding of the interactions which occur at the solid-liquid interface is vital in order to predict and recognise the subsequent biological properties of the nanoparticles both *in vivo* and *in vitro*. It is widely accepted that when nanoparticles come into contact with biological fluids a protein corona forms^7^. This is a coating that establishes around nanoparticles and is formed of two layers; a hard corona of proteins with high affinity association to the nanoparticle surface and a soft corona of proteins with a lower affinity association. In addition, biological fluids often have a high ion content and these ions will also interact with nanoparticle surfaces through electrostatic interactions^8,9^.

Nanoparticle coatings, and particularly a protein corona, are commonly analysed by electrophoresis techniques^10^. Electron microscopy (EM) has the spatial resolution for imaging and analysis of coatings on individual nanoparticles provided the specimen is appropriately prepared for the high vacuum environment of the microscope. *In vitro* testing of nanoparticles often begins by dispersion in cell culture media which is composed of a complement of amino acids, vitamins, inorganic salts, and glucose and can be supplemented with serum proteins as a source of growth factors. Clearly there is potential here for the precipitation of salts even without a serum protein supplement. Ribeiro *et al*. report the identification of the accumulation of calcium and phosphorus ions on titanium dioxide nanoparticles dispersed in cell culture media, by conventional, dry specimen STEM-EDX^11^ and also using a graphene liquid cell^12^. Xu *et al*.^13^ also report ions present in cell culture media interacting with nanoparticles; they show by analytical STEM of air dried specimens that K^+^, Ca^2+^, Na^+^, P^4−^ and Cl^−^ ions form a distinct coating around CuO and ZnO nanoparticles. These examples come from *in vitro* systems and, if correct, indicate a change in the biological identity of the dispersed nanoparticles. However, reports should give full consideration to the delicate chemistry of cell culture media and the potential that specimen preparation and analysis could have on altering this chemistry. There are a number of critical parameters that need to be considered: firstly, cell culture media contain carbonate-based buffers that maintain physiological pH only at specified CO_2_ levels (most commonly 5% CO_2_). Any changes to this CO_2_ level during incubation can result in variations in pH that change the solubility of the salts in the media. For example, one of the major manufacturers of cell culture media state that precipitation of calcium by phosphate ions will become a problem when the pH becomes more basic than 7.6^14^. Secondly, ultra-sonication is a common technique for nanoparticle dispersion but is known to cause degradation of organic molecules^15,16^ and in addition any associated temperature rise will alter the solubility of salts in the media^17^. Thirdly, drying artefacts during EM sample preparation can lead to multi-ion complexes forming around nanoparticles originally dispersed in cell culture media^18^. Therefore, *in situ* techniques such as the graphene liquid cell used by Ribeiro *et al*. in^12^ are likely to provide more accurate information about nanoparticle coatings in media. However, electron beam irradiation of liquids is well known to alter pH which could once again affect solubility of any salts in the suspending liquid^19,20^.

Any or all of these factors could potentially induce precipitation of salts in cell culture media and could lead to the artefactual formation of coatings around nanoparticles suspended in the media. Indeed, we show in this work that ion-rich coatings can be induced on a wide range of nanoparticles (Au, BaTiO_3_, ZnO, TiO_2_ and Fe_2_O_3_) of differing shape, surface charge and size when dispersed in cell culture media, and through the use of a barium titanate nanoparticle case study we identify key sources contributing to their formation. Ultimately, we provide details for appropriate specimen preparation and analysis by EM which allows analysis of nanoparticles dispersed in cell culture media free of corona artefacts.

## Methods

Nanoparticles were dispersed in de-ionised water at a concentration of 1 mg/mL using bath sonication. This was diluted in cell culture media to 100 μg/mL, either in DMEM or RPMI, both supplemented with 10% fetal bovine serum (FBS). DMEM and RPMI are common cell culture media formulated from salts, vitamins and amino acids; the two different media contain the same components but differ in the relative concentrations notably of Ca^2+^ and PO_4_^3−^ ions^8^. Henceforth, DMEM/RPMI is referred to as cell culture media (CCM) and when supplemented with 10% FBS is referred to as complete cell culture media (CCCM). A bath ultra-sonicator (VWR 80 W output) was used to disperse the nanoparticles when first suspended in the media. Transmission electron microscopy (TEM) was used to characterise the nanoparticles, in particular high angle annular dark field (HAADF) scanning TEM (STEM) imaging, energy dispersive X-ray (EDX) spectroscopy and electron energy loss spectroscopy (EELS). When necessary, on-grid blotting followed by plunge freezing and cryo-transfer to the microscope to capture and image the original dispersion of nanoparticles, in a thin layer of vitreous frozen media was used^21^. For further details regarding all methods see the supplementary information.

## Results and Discussion

**Figure 1:**
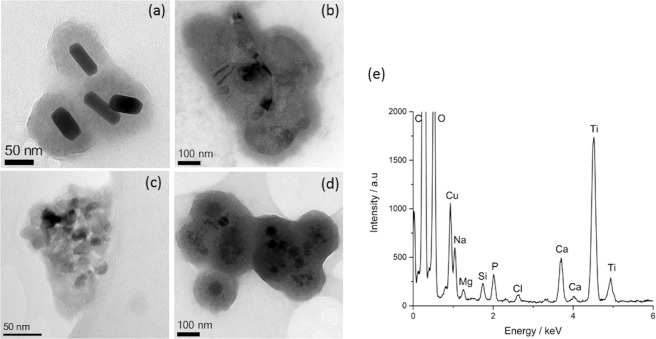
TEM images of negatively charged Au-PSS (**a**) and positively charged ZnO (**b**), TiO_2_ (**c**), and Fe_2_O_3_ (**d**) nanoparticles prepared by drop-casting sonicated dispersions of particles in CCCM onto TEM support films. A coating is seen around each different nanoparticle type regardless of surface chemistry. EDX spectroscopy indicated the presence of Ca and P in the coating around the TiO_2_ nanoparticles (**e**).

Five different nanoparticle systems were dispersed in CCCM via extended (>3 h) bath ultra-sonication, these were: Au coated with polystyrene sulfonate (Au-PSS), BaTiO_3_, ZnO, TiO_2_ and Fe_2_O_3_. All these nanoparticle types have shown promise in nanomedicine but have different structural compositions and properties including, for example, different surface charges with zeta potentials ranging from −42 mV for Au-PSS to +21 mV for Fe_2_O_3_ (Table S2 in the supplementary information). After dispersion in CCCM followed by sonication, drop-casting onto support films and air drying before transfer into the microscope we observed a coating around each type of nanoparticle by TEM (Fig. 1). The amorphous nature and composition (calcium and phosphorus rich) of this coating is similar to that reported to form around TiO_2_ nanoparticles by Ribeiro *et al*.^11,12^, and is also seen around TiO_2_ nanoparticles here (Fig. 1(C,E)). We will show however that the coating is actually an artefact of specimen preparation. To achieve this we monitored the pH of CCM during bath ultrasonication, incubation in a water bath (at 40 °C) and incubation at room temperature over a period of 24 h. Both bath ultrasonication and incubation in a water bath result in a rise of temperature to ~40 °C within 30 minutes, while there is also a significant but slower increase in pH from 7–7.25 to over 8 that takes ~120 minutes to occur (Fig. S1 in the supplementary information). There is also a pH rise of the sample incubated at room temperature, but this is even slower, taking hours to reach a value close to 8. The carbonate-based buffers present in CCM are only effective at specific CO_2_ levels, typically 5% CO_2_. If, as we did here, specimens are prepared in atmospheric CO_2_ levels (~1%) the concentration of sodium bicarbonate in the media is not correct to maintain or buffer physiological pH resulting in the recombination of HCO_3_^−^ and H^+^ to promote the production of CO_2_ and cause an increase in pH. The solubility of calcium reduces as pH increases and this change can enable the precipitation of Ca^2+^ with PO_4_^3−^ at the concentrations present in CCM^22^. We suggest that the more rapid increase in pH during bath sonication drives precipitation of calcium phosphate out of the media and onto the surface of suspended nanoparticles^23^. Furthermore, since calcium phosphate dissolution is an exothermic process this indicates that solubility decreases with increasing temperature^17^, and consequently precipitation of calcium phosphate from cell culture media is further favoured during bath sonication as the temperature rapidly increases due to excessive heating of the bulk liquid through ultrasonic waves (Fig. S1 in the supplementary information).

**Figure 2:**
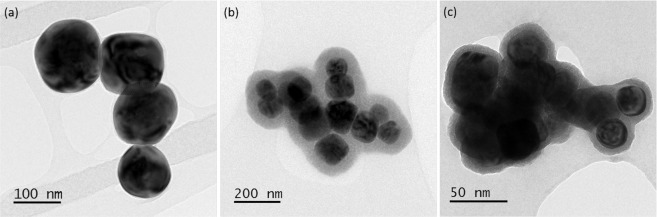
TEM images of BaTiO_3_ nanoparticles dispersed in CCCM, drop cast on TEM support films and air dried after short sonication (10 min) (**a**), prolonged sonication (>3 h) (**b**) and after placing in a water bath at 50 °C (5 h) (**c**). A significant coating was only observed after prolonged sonication or prolonged heating.

To ascertain the precise conditions under which the observed coating forms, its composition and whether it really is an artefact of sample preparation and/or analysis, we carried out a thorough investigation of BaTiO_3_ nanoparticles dispersed in CCCM. BaTiO_3_ nanoparticles were dispersed in CCCM using either a short sonication time of 10 min (Fig. 2(a)) where only a small pH change was observed or an extended sonication time of> 3 hours (Fig. 2(b)) where a significant pH increase to >8 occurred. Such extended bath sonication is required in the preparation of some nanoparticle dispersions^24,25^. We observe that a coating establishes only after extended bath sonication which suggests that the change in pH is a key factor in the mechanism of formation and from further investigation we found that the coating can actually be induced after just 30–60 minutes of sonication (Fig. S3 in the supplementary information). We confirmed that an incubation time of a few hours was not the critical factor by observing no coating on a sample left at room temperature for 3 h (Fig. S2 in the supplementary information). In addition it is the pH rise induced by the temperature change rather than the physical sonication that induces a coating since a coating still established around BaTiO_3_ particles dispersed in CCCM when incubated in a water bath at >40 °C for >3 hours without ultrasonication (Fig. 2(c)). A similar rate and amount of increase in pH was observed during water bath incubation at 40 °C to that seen in bath sonication (Fig. S1 in the supplementary information). Finally, we ascertained that the presence or otherwise of FBS in the media did not prevent or promote coating formation, further confirming that the coating is ionic and independent of any protein absorption.

**Figure 3:**
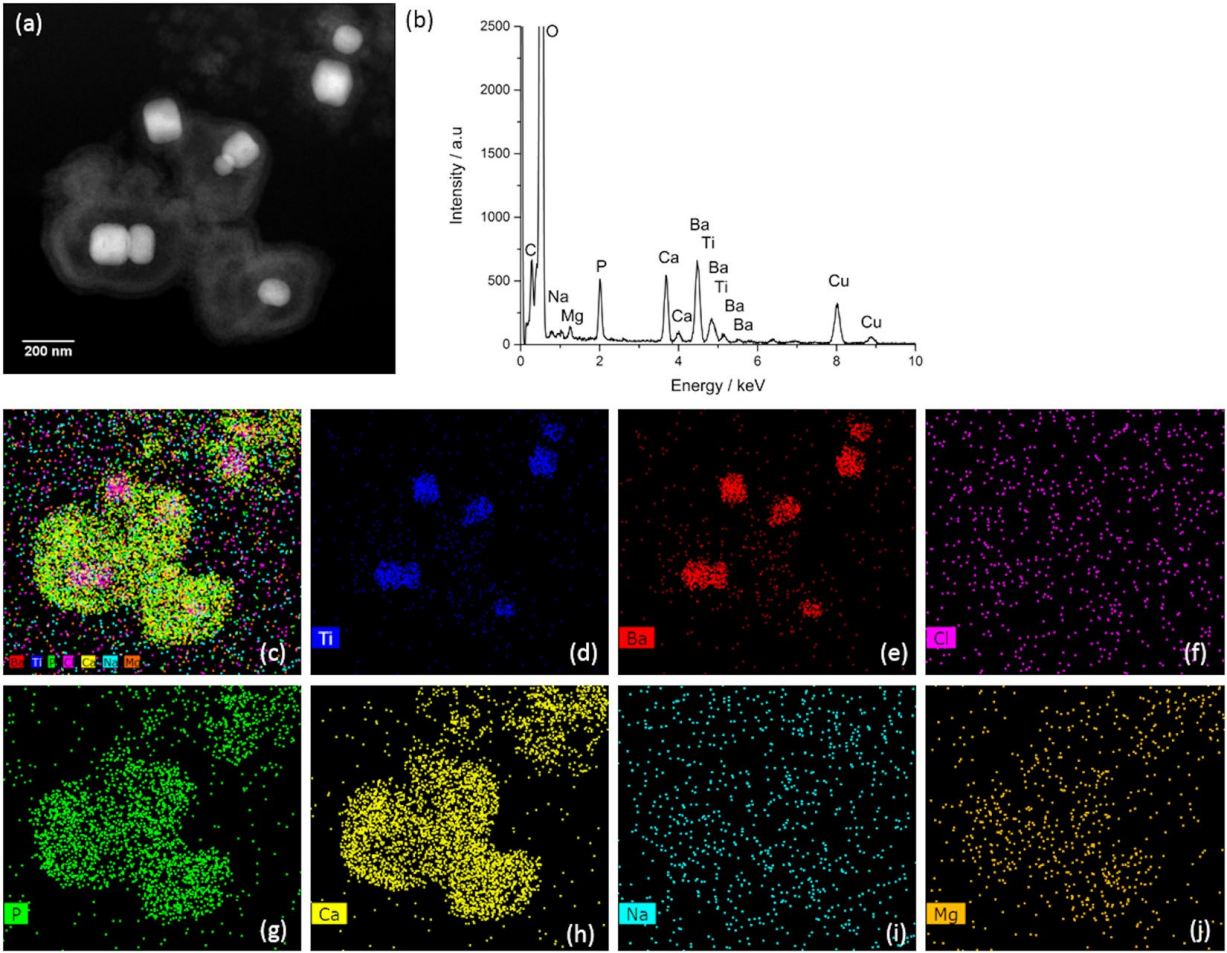
(**a**) Cryo-HAADF STEM image of BaTiO_3_-PLL nanoparticles dispersed in frozen, vitrified CCCM prepared via extended bath sonication (>3 h). EDX signals were obtained indicating the presence of Ti (**d**) and Ba (**e**) along with components of the media; Cl (**f**), P (**g**), Ca (**h**), Na (**i**), Mg (**j**). The EDX maps indicate the frozen hydrated coating is a calcium-phosphorus-rich corona with less ion content from the other components of the media than an air-dried coating (c – combined EDX maps).

Previous work has shown conventional drop-cast, air dried TEM sample preparation of sonicated BaTiO_3_ nanoparticles dispersed in CCCM can be affected by drying artefacts, whereby other ions in the media such as Na^+^, Cl^−^ and K^+^, also precipitate in the coating^18^. We further confirm this using two other nanoparticle systems of ZnO and Fe_2_O_3_ (Fig. S5 in the supplementary information). We suggest such artefacts could lead to the multi-ion complex reported to form around ZnO and CuO nanoparticles dispersed in CCCM by Xu *et al*.^13^. To eliminate such drying artefacts nanoparticles can be imaged in the frozen hydrated state by blotting and plunge freezing a nanoparticle suspension on a TEM grid into liquid ethane and transferring this into the microscope to be analysed frozen^26^. Cryo-analytical-STEM of sonicated BaTiO_3_ nanoparticles prepared in this way shows a coating significantly richer in Ca and P than when air dried (Fig. S6 in the supplementary information). More significantly elemental mapping confirms a coating similar in size, form and chemistry to that reported by Ribeiro *et al*.^11,12^ BaTiO_3_ nanoparticles have a negative surface charge, yet often in *in vitro* studies the chemistry of a nanoparticle surface is altered to be positively charged to enhance cellular uptake^27^. As such, we coated BaTiO_3_ nanoparticles with poly-l-lysine (BaTiO_3_-PLL) and, upon suspending the BaTiO_3_-PLL nanoparticles in CCCM and sonication in atmospheric conditions we observed the same calcium phosphate based coating around the nanoparticles (Fig. 3).

Selected area electron diffraction (SAED) indicated that the coating was amorphous. To determine the composition of the coating we used EDX and EELS quantification benchmarked against calcium phosphate standards (Table S1 in the supplementary information). From EDX quantification the average Ca/P ratio of the coating that formed around BaTiO_3_ in CCCM was 1.39 ± 0.30. From EELS quantification the Ca/P ratio for BaTiO_3_ in CCCM was 1.63 ± 0.46 (Table S3 in the supplementary information). The reported error is taken from the standard deviation of n > 3 measurements. Any of octacalcium phosphate, tri basic calcium phosphate and hydroxyapatite (HA) have a composition close to these values with Ca/P ratios of 1.33, 1.5 and 1.67 respectively. To narrow this further we used thermodynamic modelling to predict the precipitation of calcium phosphate from a system modelled on the ions present in DMEM (HSC Chemistry, v 5.1^28^). This predicted that the most favoured precipitate was HA and consequently we suggest the most likely form of the observed calcium phosphate coating is an amorphous calcium phosphate of HA stoichiometry.

We believe unintentional induction of a calcium phosphate coating could have significant implications for the interpretation of cell uptake and toxicological studies. Knowledge of the protein corona has helped the design of nanoparticles for use in medicine by establishing which coatings can prevent or promote protein binding^29,30^. Calcium can act as a bridging agent for the adsorption of negatively charged species such as bovine serum albumin (often a large component of protein coronas) onto nanoparticles with a negatively charged surface^31,32^ and differences in protein adsorption to calcium phosphate coated surfaces have also been detailed^33^. Furthermore, some surface coatings are designed to improve colloidal stability of nanoparticles, and an additional layer of calcium phosphate may disrupt this and alter the dispersion of the particles. At the very least the corona will be altered if calcium phosphate competes for binding at surfaces of nanoparticles and this may affect the biological function of the nanoparticles, as suggested by Riberio *et al*.^12^. This would however only be the case if all the nanoparticles for cellular delivery were inappropriately prepared rather than just those for STEM investigation. If this were the case this would also require a further pH adjustment of the modified suspension, back to physiological levels, prior to delivery to cells.

To prevent future reports of calcium phosphate coatings on nanoparticles dispersed in CCM and CCCM that are potentially only artefacts of the specimen preparation or analysis method used for EM analysis, we put forward a number of recommendations in order to minimise this occurrence:

- When using cell culture media for specimen preparation we recommend monitoring the pH to ensure no significant changes are occurring, and to work at as close to physiological conditions as possible. For example, if prolonged sonication is required for nanoparticle dispersion, this should be carried out in water before diluting down to appropriate concentrations in the required biological media.
- Cryo-S/TEM analysis eliminates any drying artefacts and, at an appropriate electron fluence, can reduce some of the irradiation-induced artefacts that can occur in liquid cell TEM analysis^26^.
- In research collaborations where research is undertaken in multiple locations, clear and precise communication between all research facilities is essential to ensure exact procedures are replicated in all locations.

**Figure 4:**
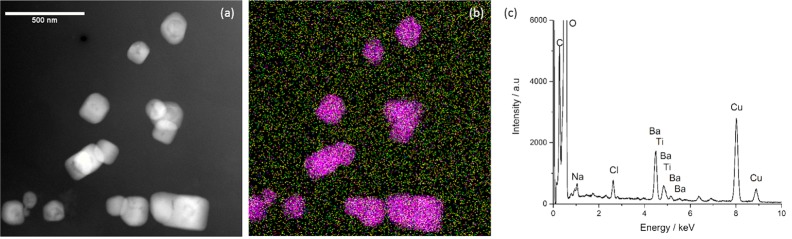
(**a**) Cryo-HAADF STEM image of BaTiO_3_-PLL nanoparticles dispersed CCCM prepared by sonication in water and then dilution in CCCM before vitrification and transfer to the microscope. There is no evidence of a calcium phosphate coating around the nanoparticles. This is confirmed by Cryo-EDX spectroscopy (**c**) which shows signals only for Ba and Ti from the nanoparticles, Cu from the grid and Na and Cl present throughout the section due to salt within CCCM. The combined elemental map in (**b**) maps the Ba (red), Ti (blue), Na (green) and Cl (yellow) signals.

We have shown that carefully implementing these recommendations on a sample of BaTiO_3_-PLL nanoparticles dispersed in CCCM and imaged in the frozen hydrated state does not result in the formation of an amorphous calcium phosphate coating (Fig. 4). Here, nanoparticle dispersion was carried out via extended bath sonication of the particles dispersed in water before diluting in CCCM without further sonication. Therefore the pH of the CCCM dispersion did not change significantly during specimen preparation and the sample was maintained at room temperature prior to plunge freezing. These precautions successfully removed any induced precipitation onto the nanoparticles. There are also options to use a probe sonicator as an alternative to bath sonication. This would provide more power so reducing the time available for pH changes to be established. However, this recommendation comes with caution as the higher power carries an increased risk of detrimentally causing physical or chemical changes to the inherent properties of the nanoparticles or media within a suspension^34^.

In summary, we have shown that specimen preparation for EM analysis of nanoparticles dispersed in cell culture media without due care to maintain physiological pH can induce a calcium phosphate rich, amorphous coating around the nanoparticles. This coating is independent of nanoparticle material or size and forms upon a significant rise in pH beyond physiological levels to 8.0 (driven in our case by 30–60 min of bath sonication in atmospheric CO_2_ levels) and may be misinterpreted as a genuine feature of dispersion of the nanoparticles in cell culture media. We demonstrate that specimen preparation without any change in pH of the dispersing media results in no indication of an ionic coating. We recommend that specimen preparation protocols for EM are therefore checked and monitored for any procedures that could cause such detrimental artefacts.

## Supplementary Information

Supplementary Information.
