# Supplementary material for: Nanoparticle corona artefacts derived from specimen preparation of particle suspensions
Source: Sci Rep. 2020 Mar 24;10:5278. doi: 10.1038/s41598-020-62253-y (PMC7093507; doi:10.1038/s41598-020-62253-y)
Supplement: Supplementary file 1 — Supplementary Information. [file 41598_2020_62253_MOESM1_ESM.pdf]

Nanoparticle corona artefacts derived from specimen preparation of particle suspensions

Martha Ilett[1], Omar Matar[1], Faith Bamiduro[1], Sergio Sanchez-Segado[2], Rik Brydson[1], Andy Brown[1] and Nicole Hondow[1]*

[1]School of Chemical and Process Engineering, University of Leeds, Leeds, UK.

[2]Department of Chemical and Environmental Engineering, Technical University of Cartagena, Campus Muralla del Mar C/Doctor Fleming s/n, Cartagena, 30202, Murcia, Spain.

SUPPLEMENTARY INFORMATION

1. Methods

1.1 Nanoparticle synthesis

$BaTiO_3$ nanoparticles were synthesised at 150°C for 72 h via a hydrothermal method with particles of diameter 130 nm obtained.[1,2] Similarly ZnO nanoparticles were synthesised via a hydrothermal method at 120°C for 12 h.[3] Three different sized nanoparticles were synthesised by altering the pH of the reaction solution using NaOH pellets (Reidel-de Haen, Lot# 60100).[4] The pH of the precursor solution was altered as follows: ZnO-A 9.43 (desired 9), ZnO-B 11.08 (desired 11) and ZnO-C pH 12.9 (desired 13). The three sizes measured from the longest length were ZnO-A 840 nm, ZnO-B 400 nm and ZnO-C 150 nm. Fe_2O_3 nanoparticles <50 nm were purchased from Sigma Aldrich (Lot# 40095/1 22/00). Gold nanoparticles coated with polystyrene sulfate were synthesised via the methods described in [5,6] and were kindly donated by Lucien Roach of the Molecular and Nanoscale Physics group, School of Physics and Astronomy, University of Leeds.

1.2 Transmission Electron Microscopy

Transmission electron microscopy (TEM) was undertaken using two microscopes; an FEI Tecnai F20 operated at 200 kV and fitted with a Gatan Orius CCD camera and Oxford instruments 80 mm^2 X-max EDX detector and an FEI Titan[3] Themis G2 operating at 300 kV fitted with 4 EDX silicon drift detectors, a Gatan One-View CCD and a Gatan Quantum 965 ER imaging filter.

For conventional drop-cast TEM, holey carbon film copper grids were plasma treated under argon/10% O_2 onto which the nanoparticle dispersion was drop cast and left to dry in air for several minutes. For cryo-TEM sample preparation an FEI Mark IV Vitrobot© was used. A 3.5 µl drop of sample was placed on a Quantifoil (Ra1.2/1.3; EM Resolutions) or Lacey carbon (EM resolutions) grid, blotted and rapidly plunged into liquid ethane. The grid was transferred to the microscope using a Gatan 914 TEM cryo-holder and maintained at a temperature of <-165°C.

For EDX spectroscopy, Oxford instruments Aztec software and Bruker Espirit software was used. Dual-EELS analysis was carried out using collection and convergence semi-angles of 21 and 8.2 mrad respectively. For all cryo-analytical STEM, a probe current between 40 and 100 pA was used and for EDX a dwell time of 23 µs. For cryo-EELS, to prevent destruction of the vitreous ice during acquisition the electron beam was continually scanned across the image area (5 µs dwell time) and the EEL spectra obtained for the whole area.[7] In all instances the total accumulated fluence used was below the threshold for significant compositional change of hydroxyapatite reported as 100 x 10^6 e^-/nm^2 by Eddisford *et al.*[8]

For Ca and P semi-quantification for EDX the K_α X-ray lines were used for both Ca and P at 3.69 and 2.013 keV respectively and for EELS the $L_{2,3}$ edges were used for both Ca and P. Reference standards of tribasic calcium phosphate (Fluka Chemika) and hydroxyapatite (Sigma Aldrich) were used to ensure accurate quantification and the results are shown in table S1.

Table S1: Standard calcium phosphates of tribasic and hydroxyapatite were used to confirm accurate relative semi-quantification of the calcium to phosphorus ratio using EDX and EEL spectroscopy. Errors were calculated as the standard deviation of n>3 measurements.

	EDX	EELS	Expected
Tribasic calcium phosphate	1.42 ± 0.03	1.48 ± 0.23	1.5
Hydroxyapatite	1.60 ± 0.04	1.77 ± 0.14	1.67

2. Supplementary results

2.1 Nanoparticle surface charge

Table S2: Zeta potential measurements for 6 different nanoparticles. Measurements were taken from dispersions in water and averaged from n=3.

	Zeta Potential (mV)
Au-PSS	- 42 ± 0.6
BaTiO3	- 24 ± 0.8
ZnO	+ 7.5 ± 0.6
TiO_2	+ 11 ± 0.6
Fe_2O_3	+ 21 ± 0.3
$BaTiO_3$-PLL	+ 48 ± 0.3

2.2 pH and temperature change

pH and temperature measurements were taken of cell culture media either bath sonicated, incubated in a water bath at 40°C or incubated at room temperature (Figure S1).

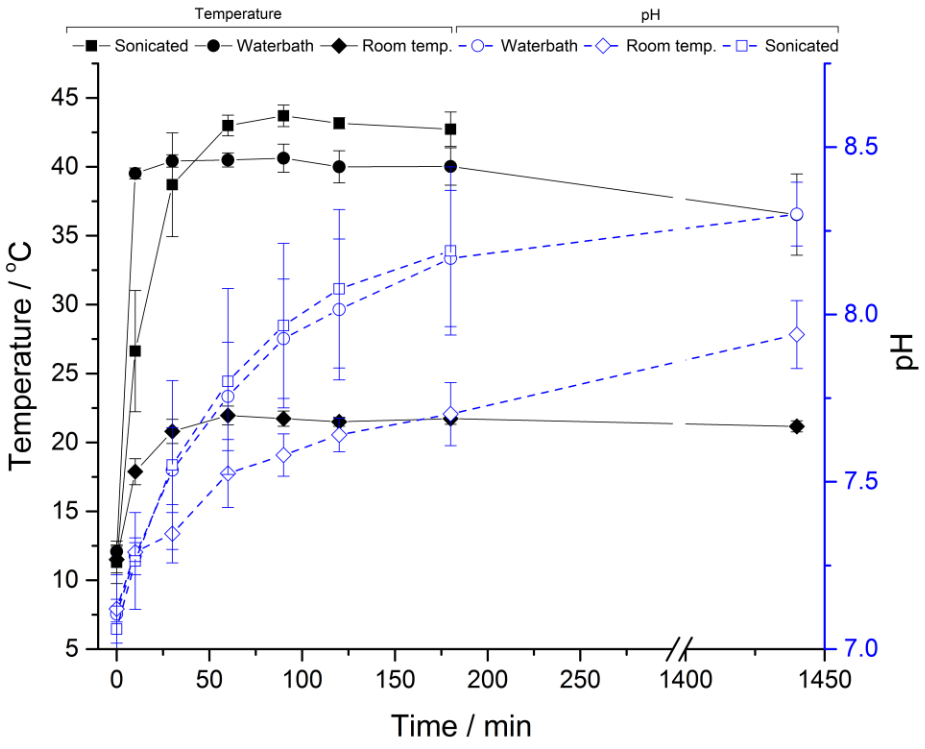

Figure S1: pH (dashed line) and temperature (solid line) measurements of cell culture media either bath sonicated, incubated in a water bath at 40°C or incubated at room temperature. For bath sonication, measurements were taken over a period of 3 h; whilst for incubation at 40°C in a water bath and at room temperature, measurements were taken over a period of 24 h. The increase in pH of the cell culture media was much quicker when at the elevated temperatures of the water bath and bath sonicator reaching >8 after 3 h. In comparison the increase in pH at room temperature was much slower, only reaching ~8 after 24 h incubation.

To confirm that the pH and temperature change were the critical factors in the formation of a calcium phosphate coating rather than incubation time, $BaTiO_3$ nanoparticles were dispersed in cell culture media for 3 h at room temperature. Figure S2 shows there was no evidence of a calcium phosphate coating around the nanoparticles.

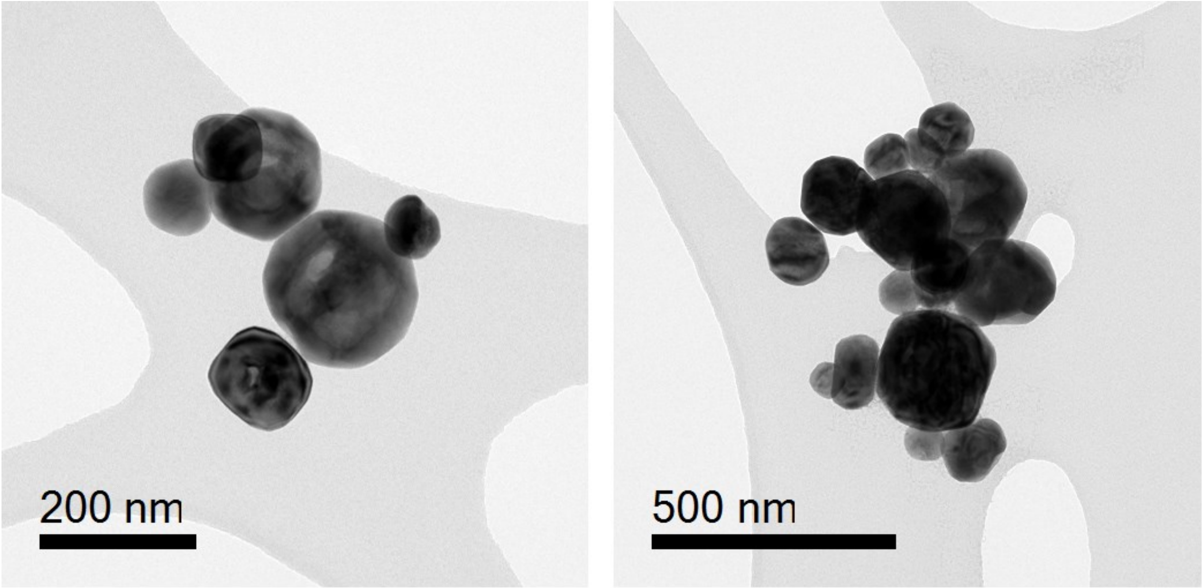

Figure S2: $BaTiO_3$ nanoparticles dispersed in cell culture media and incubated at room temperature for 3 h. No calcium phosphate coating is observed, confirming that pH and temperature change are the critical factors in coating formation.

2.3 Sonication time series

The calcium-phosphorus-rich coating was monitored after progressive sonication times indicating that the coating establishes after just 30-60 minutes of sonication (Figure S3).

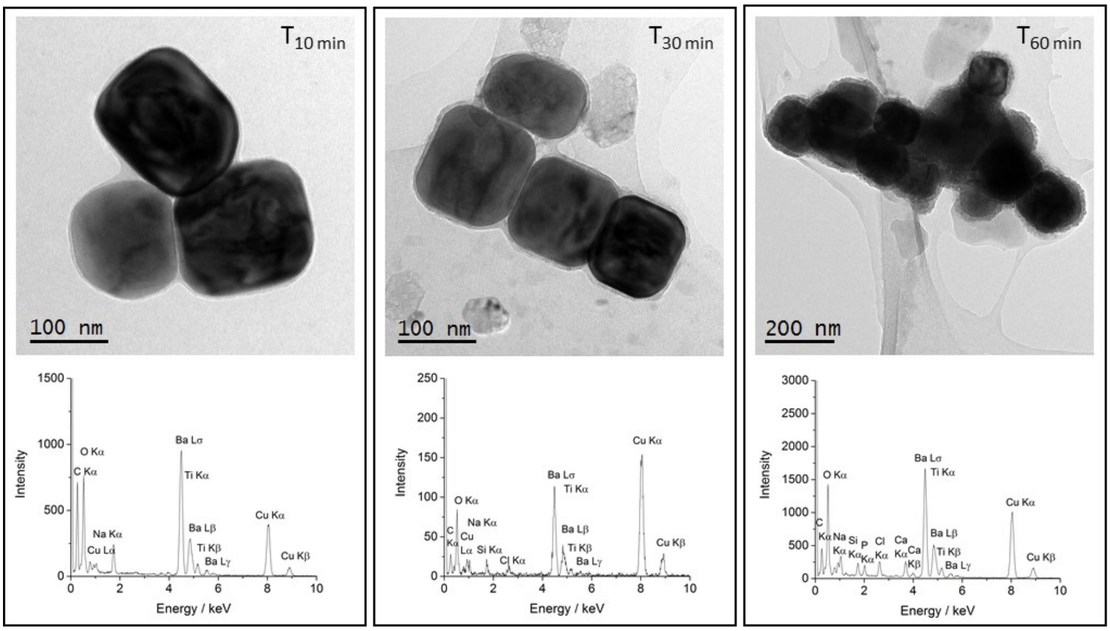

Figure S3: Time study of the formation of a calcium phosphate coating around $BaTiO_3$ nanoparticles. The calcium phosphate coating established after 1 h. Na and Cl are attributed to the formation of salts some of which can be seen in the TEM images at T_{30min} and T_{60min} in addition to the drying artefacts previously described in.[9]

2.4 Nanoparticle size

Three ZnO nanoparticles of average diameter 840 nm (ZnO-A), 400 nm (ZnO-B) and 150 nm (ZnO-C) were dispersed in CCCM at an equal weight per volume of 100 µg/mL. Following extensive bath sonication, a coating was evident on all three particles, which was confirmed as Ca and P rich through EDX analysis (data not shown). No significant different between the thickness of the three coatings was observed with average calcium phosphate coating thicknesses of 65 (±17) nm, 49 (±8) nm and 46 (±13) nm for ZnO-A, ZnO-B and ZnO-C respectively (Figure S4).

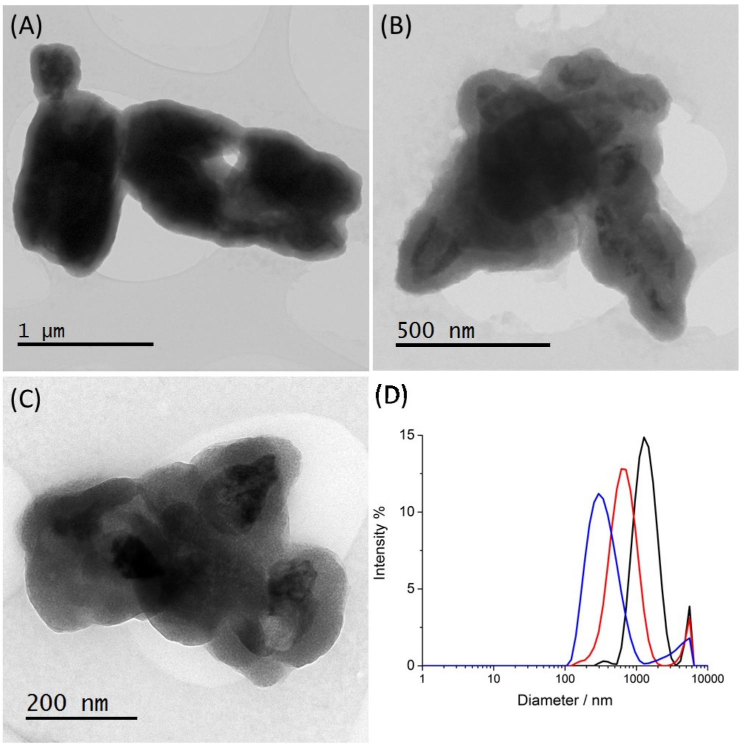

Figure S4: TEM images of ZnO nanoparticles of varying size and shape dispersed in CCCM prepared via drop casting. Nanoparticle primary particle size increased with decreasing precursor pH during synthesis. This is confirmed in the DLS plot (D) where there is an increase in size between ZnO-A (blue), ZnO-B (red) and ZnO-C (black). All three dispersions showed a calcium phosphate coating could be formed around the nanoparticles independent of size and/or shape.

2.5 Cryo-analytical STEM

Confirmation that using *in situ* cryogenic techniques eradicates drying artefacts (previously described in Ilett *et al.*[9]) was demonstrated by cryo-analytical STEM of ZnO and Fe_2O_3 nanoparticles dispersed in CCCM by sonication (Figure S5) and $BaTiO_3$ (Figure S6).

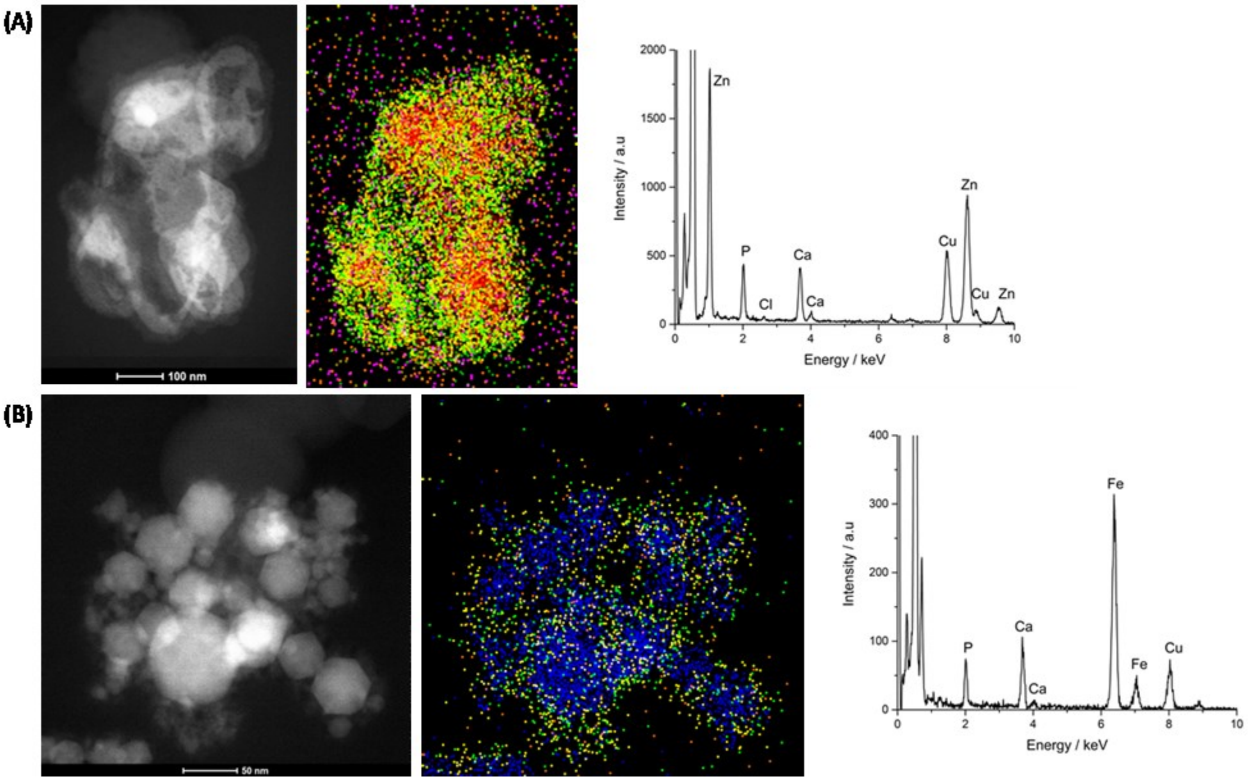

Figure S5: ZnO (A) and Fe_2O_3 (B) nanoparticles dispersed in CCCM, sonicated, blotted and plunge frozen to capture in a layer of vitreous CCCM. For both nanoparticle systems a Ca and P rich coating was observed with signals for other components in the media spatially resolving as in solution. Combined EDX maps are show with Zn (red), Ca (yellow), P (green), Cl (pink) and Fe (blue).

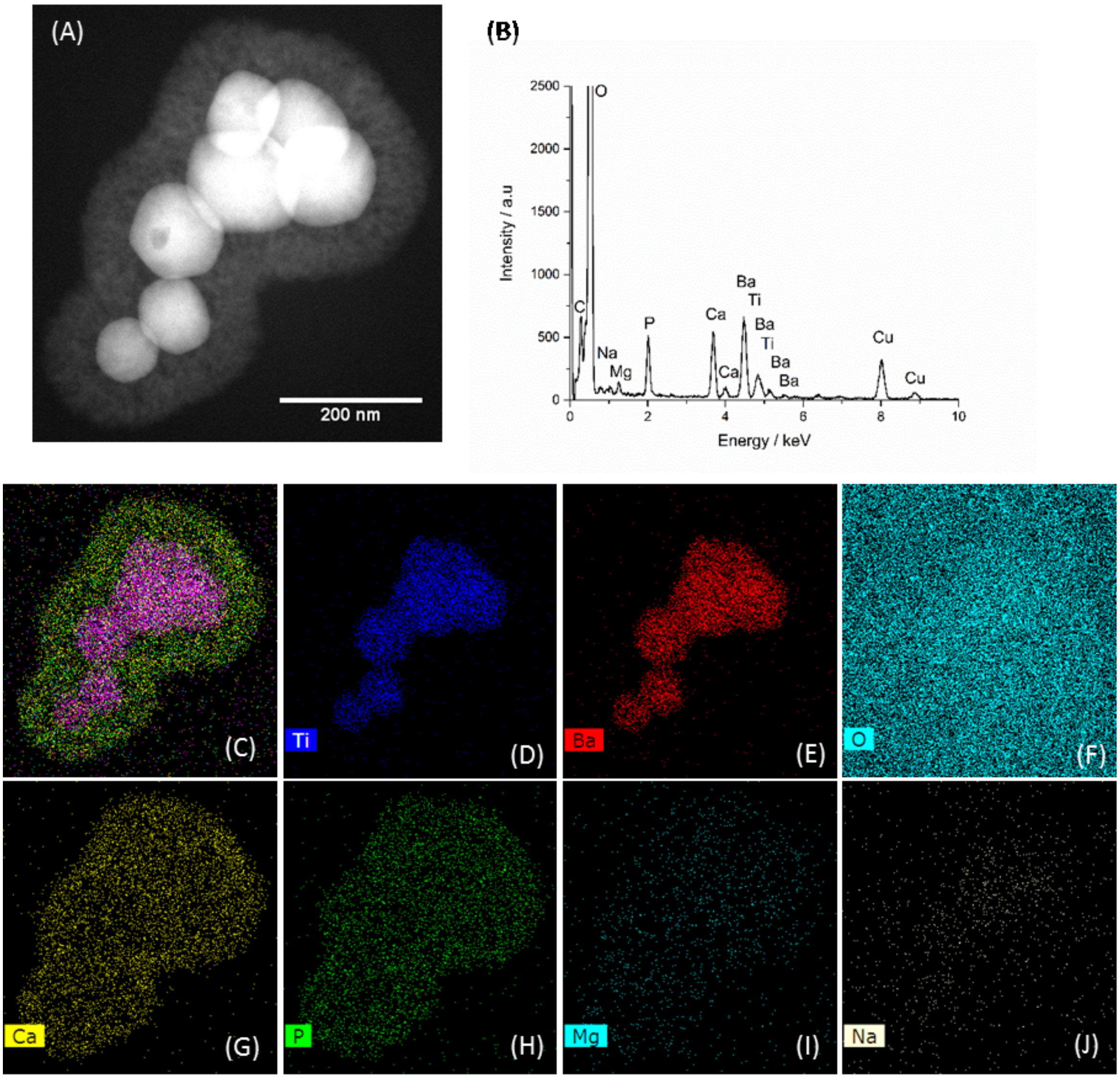

Figure S6: (A) Cryo-HAADF STEM image of $BaTiO_3$ nanoparticles dispersed in CCCM prepared via extended bath sonication (>3 h). EDX signals indicated the presence of Ti (D) and Ba (E) along with components from the media; Ca (G), P (H), Mg (I), Na (J). Spatially resolving the position of the elements indicated that the coating is calcium and phosphorus rich.

2.6 Ca/P ratio quantification

The compositions and in particular the concentrations of Ca^{2+} and PO_4^{3-} vary significantly between DMEM and RPMI with each having Ca^{2+} and PO_4^{3-} concentrations of 1.8 mM and 0.92 mM (DMEM) and 0.42 mM and 5.63 mM (RPMI) respectively.[10] However a calcium phosphate coating was observed for $BaTiO_3$ and $BaTiO_3$-PLL nanoparticles dispersed in both media after >1 h bath sonication and, although there was a suggestion from EDX relative semi-quantification that there may be a reduced Ca/P ratio when using RPMI, this was not significant when standard deviations were taken into account (Table S3). Since the calcium phosphate coating established around nanoparticles

suspended in either media this would suggest that it is changes in solubility that is the key factor in coating formation rather than the absolute levels of calcium and phosphorus present in solution.

Table S3: EDX and EELS quantification of the Ca to P ratio in the calcium phosphate coating around PLL coated and uncoated $BaTiO_3$ nanoparticles dispersed in either DMEM or RPMI (both with 10% FBS supplement). The ratios were calculated from different sites in a number of samples using Bruker Espirt software (EDX) and Gatan Digital Micrograph (EELS).

		EDX analysis			EELS analysis
		$BaTiO_3$ in DMEM	$BaTiO_3$-PLL in DMEM	BaTiO3-PLL in RPMI	$BaTiO_3$ in DMEM
**Sample 1**	Site 1	1.44	1.15	1.21	1.52
	Site 2	1.39	1.34	-	1.03
	Site 3	1.18	1.19	-	1.76
	Site 4	-	2.69	-	1.52
	Site 5	-	1.14	-	2.67
	Site 6			-	1.86
**Sample 2**	Site 1	1.21	1.34	1.11	1.32
	Site 2	1.15	1.26	-	1.58
	Site 3	-	1.35	-	1.38
	Site 4	-	1.27	-	-
	Site 5	-	1.10	-	-
**Sample 3**	Site 1	1.94	-	1.22	-
**Average**		**1.39±0.30**	**1.38±0.47**	**1.21±0.06**	**1.63±0.46**

References

1 Eckert, J. O., Hung-Houston, C. C., Gersten, B. L., Lencka, M. M. & Riman, R. E. Kinetics and Mechanisms of Hydrothermal Synthesis of Barium Titanate. *J. Am. Ceram. Soc.* **79**, 2929-2939, doi:10.1111/j.1151-2916.1996.tb08728.x (1996).

2 Matar, O. *et al.* Barium Titanate Nanoparticles for Biomarker Applications. *Journal of Physics: Conference Series* **644**, 012037 (2015).

3 Bamiduro, F., Ward, M. B., Brydson, R. & Milne, S. J. Hierarchical Growth of ZnO Particles by a Hydrothermal Route. *J. Am. Ceram. Soc.* **97**, 1619-1624, doi:10.1111/jace.12809 (2014).

4 Wang, H., Xie, J., Yan, K. & Duan, M. Growth Mechanism of Different Morphologies of ZnO Crystals Prepared by Hydrothermal Method. *Journal of Materials Science & Technology* **27**, 153-158, doi:https://doi.org/10.1016/S1005-0302(11)60041-8 (2011).

5 Roach, L. *et al.* Morphological control of seedlessly-synthesized gold nanorods using binary surfactants. *Nanotechnology* **29**, 135601, doi:10.1088/1361-6528/aaa99d (2018).

6 Leonov, A. P. *et al.* Detoxification of gold nanorods by treatment with polystyrenesulfonate. *ACS Nano* **2**, 2481-2488, doi:10.1021/nn800466c (2008).

7 Ilett, M., Brydson, R., Brown, A. & Hondow, N. Cryo-analytical STEM of frozen, aqueous dispersions of nanoparticles. *Micron* **120**, 35-42, doi:10.1016/j.micron.2019.01.013 (2019).

8 Eddisford, P., Brown, A. & Brydson, R. Identifying and quantifying the mechanism of electron beam induced damage and recovery in hydroxyapatite. *Journal of Physics: Conference Series* **126**, 012008, doi:10.1088/1742-6596/126/1/012008 (2008).

9 Ilett, M. *et al.* Cryo-STEM-EDX spectroscopy for the characterisation of nanoparticles in cell culture media. *J. Phys. Conf. Ser* **902**, 012006 (2017).

10 Moore, T. L. *et al.* Nanoparticle colloidal stability in cell culture media and impact on cellular interactions. *Chem. Soc. Rev.* **44**, 6287-6305, doi:10.1039/c4cs00487f (2015).
